# Reconceptualising Rural Cancer Inequalities: Time for a New Research Agenda

**DOI:** 10.3390/ijerph17041455

**Published:** 2020-02-24

**Authors:** Christina Dobson, Greg Rubin, Peter Murchie, Sara Macdonald, Linda Sharp

**Affiliations:** 1 Population Health Sciences Institute, Newcastle University, Sir James Spence Institute, Royal Victoria Infirmary, Newcastle-upon-Tyne NE1 4LP, UK; gregory.rubin@newcastle.ac.uk (G.R.); linda.sharp@newcastle.ac.uk (L.S.); 2 Division of Applied Health Sciences, Section of Academic Primary Care; University of Aberdeen, Polwarth Building, Foresterhill, Aberdeen AB25 2ZD, UK; p.murchie@abdn.ac.uk; 3 Institute of Health Sciences, General Practice & Primary Care; 1 Horselethill Road, Glasgow G12 9LX, UK; sara.macdonald@glasgow.ac.uk

**Keywords:** cancer survival, diagnostic pathways, symptoms, treatment, rural populations, inequalities

## Abstract

Evidence has shown for over 20 years that patients residing in rural areas face poorer outcomes for cancer. The inequalities in survival that rural cancer patients face are observed throughout the developed world, yet this issue remains under-examined and unexplained. There is evidence to suggest that rural patients are more likely to be diagnosed as a result of an emergency presentation and that rural patients may take longer to seek help for symptoms. However, research to date has been predominantly epidemiological, providing us with an understanding of what is occurring in these populations, yet failing to explain why. In this paper we outline the problems inherent in current research approaches to rural cancer inequalities, namely how ‘cancer symptoms’ are conceived of and examined, and the propensity towards a reductionist approach to rural environments and populations, which fails to account for their heterogeneity. We advocate for a revised rural cancer inequalities research agenda, built upon in-depth, community-based examinations of rural patients’ experiences across the cancer pathway, which takes into account both the micro and macro factors which exert influence on these experiences, in order to develop meaningful interventions to improve cancer outcomes for rural populations.

## 1. Introduction

Rural environments are often conceived of as places that both create and sustain healthy bodies, and in which healthy bodies can belong and flourish [1,2]. We have known for almost 30 years, however, that people living in rural areas have poorer survival than their urban counterparts for a number of cancers [3]. This phenomenon has been observed throughout the developed world, yet remains largely unexplained.

Rural cancer patients have been found to have more advanced disease at diagnosis in some studies [4,5,6] and are more likely to die before their disease is diagnosed [7,8], suggesting that long times to diagnosis may be a characteristic of this population. However, the evidence base is not entirely clear, with other work suggesting that some of the most remote cancer patients have less advanced disease at diagnosis [9]. Recent Danish data shows that rural cancer patients have more advanced disease for ‘easy to diagnose’ cancers and less advanced disease for ‘hard to diagnose’ cancers [10,11]. The route through which someone is diagnosed influences their likelihood of survival, with diagnosis via emergency presentation associated with some of the poorest outcomes, and diagnosis via screening some of the best [12]. People living in rural areas are more likely to be diagnosed as the result of an emergency presentation and less likely to be diagnosed via screening than people living in urban areas [8], with uptake of screening believed to be impacted by the longer travel time to health care services that rural residents face [3].

How long it takes someone to be diagnosed with cancer, after the emergence of symptoms, is associated with their chance of survival [13]. The cancer diagnostic pathway is made up of a series of stages: the ‘patient interval’ (time from symptom onset to first presentation), ‘primary care interval’ (time from first presentation to a health care practitioner to specialist referral) and the ‘secondary care interval’ (time from specialist referral to diagnosis), with progress through these intervals shaped by a range of contextual factors which vary between individuals and populations [14,15,16]. Whether these intervals, markers of their duration (such as disease stage at diagnosis), or the routes that people follow to diagnosis significantly differ for rural and urban cancer patients remains unclear.

Lung cancer patients in rural Western Australia were found to take longer to seek help for symptoms [17], with the impact of travel time to General Practitioner (GP) practices and rural Australians’ constructions of stoicism and machismo possibly contributing to the length of rural cancer patients’ patient intervals [18]. Amongst Scottish cancer patients, there has been found to be an association between rurality and diagnosis via emergency presentation [8], suggesting that rural patients may wait longer before seeking help for their symptoms than people in urban areas do.

However, as is the case with the evidence on rurality and stage at diagnosis, data on rurality and diagnostic intervals presents a similarly complex picture. For instance, rural colorectal cancer patients in Western Australia seem to experience the greatest diagnostic delays after first presentation with symptoms, suggesting that delays may be occurring within the health care system intervals of the diagnostic pathway for these patients [19]. Once patients are referred from primary care into secondary care for specialist assessment of their symptoms, rural patients may be diagnosed and treated just as quickly, if not sooner, than urban patients [9,20], suggesting that diagnostic delays may be more prevalent in the patient and primary care intervals. It may be that there is something inherently ‘different’ about the way rural patients experience and navigate the patient and primary care intervals when compared to their urban counterparts. For instance, rural patients may take longer to consult because of a preference to visit their own GP [21], or because they are faced with more competing priorities than urban patients [22]. Alternatively, perhaps rural GPs have inherently ‘different’ relationships with their patients compared to GPs in urban areas, which may affect management in primary care and onwards referral [23].

Another possibility is that factors which occur post-diagnosis may be contributing to the poorer survival in rural populations. There is evidence to suggest that rural cancer patients may be less likely to undergo cancer-directed surgery or receive radiotherapy or chemotherapy [3]. Treatment receipt tends to be associated with better survival [24,25,26] and treatment in a higher volume hospital has been shown to be associated with better outcomes following cancer-directed surgery [27,28]. Since larger hospitals tend to be located in urban areas, it may be that rural patients are less likely to be treated in higher volume hospitals. They may also be faced with more complex decisions about which treating facility to attend, seeking to balance personal, social and logistical factors with perceived quality of accessible care [29]. We know that rural patients generally have longer to travel to a specialist cancer centre (something which has stimulated the development of teleoncology initiatives [30]) and this, or the likely duration of time away from home, may affect the treatment options that these patients have, or the choices they make [31]. Similar factors may affect likelihood of completing a course of radiotherapy or chemotherapy.

Given that a significant proportion of people with cancer experience a subsequent primary tumour [32,33] (which also adversely impacts survival [34,35]), and that there is evidence to suggest that rural patients may take longer to seek help for initial symptoms, it could be that rural patients also take longer to seek help for symptoms of a subsequent cancer or cancer recurrence, thus affecting survival. However, these hypotheses remain just that, given the absence of detailed, in depth research into the stages of the diagnostic pathway and post-diagnosis experiences of rural cancer patients.

Approaches to examining rural cancer inequalities have varied very little over the past 20 years, despite having two inherent weaknesses. Firstly, they generally depart from the standpoint that people experience and appraise symptoms with ‘cancer’ in mind. Secondly, ‘rural’ and ‘urban’ are largely defined using categorical variables, based on a characteristic of the area, such as population density. These foundational assumptions are problematic, as they fail to account for the complexity of rural patients’ experiences and how these are shaped and governed by rural environments. This over-simplification of both ‘cancer symptoms’ and ‘the rural’ may be hindering our efforts to understand and address the inequalities that rural cancer patients experience.

In the remainder of this article we outline the problems inherent in current treatment of the concepts of ‘rurality’ and ‘cancer symptoms’ and call for a revised research agenda which reconceptualises rurality in relation to cancer inequalities. We argue for the need to develop and implement novel ways of examining rural cancer inequalities, by working with rural communities to understand how embodied experience, help-seeking, diagnostic experiences and experiences later in the cancer pathway are created and shaped by the rural environments in which they occur. This requires the research community to move beyond the quantitative studies, often of routinely-collected health service data, that have dominated this research field to develop detailed, in depth knowledge of the lived experiences of rural cancer patients and the contexts within which these take place. By using qualitative methods, such as interviewing and ethnography, we can begin to understand the geographically-, culturally- and socially-located experiences of rural cancer patients, in order to identify and address the root causes of the health inequalities they experience.

## 2. Symptomatic Experience

Researchers and clinicians routinely talk about a person’s appraisal of, and help-seeking for, cancer symptoms. However, for the majority of individuals the experience, management and responses to symptoms precede any notion of, or association with, cancer. In fact, symptoms, including those that may herald a cancer, are routinely experienced within the general population. Many of these are never reported to primary care, a phenomenon known as the ’symptom iceberg’ [36]. Indeed most of the symptoms that are reported to a health care practitioner will turn out to be benign. People’s initial appraisal of an unusual bodily sensation does not always include consideration of underlying aetiology, as not every sensation we experience will come to be conceived of as a symptom. Of those sensations that do become perceived of as symptoms, cancer may be only one of a number of underlying causes considered by the individual, if at all. Investigating how and when people decide to seek help for symptoms that may be caused by cancer is vital. However, we must be mindful of how we frame and investigate the experiences of people with potential cancer symptoms, as their experience, appraisal and response to these symptoms is often done without reference to cancer as a personally-relevant disease. In order to effectively understand how rural populations experience and manage cancer symptoms, we therefore need to understand how people residing in rural areas experience, define and manage abnormal bodily sensations, taking into account how local environments, communities, lifestyles, responsibilities and ability to access services shape these experiences.

Research in the fields of medical anthropology and sociology highlights how abnormal bodily sensations are often the first indication to an individual that something may be ‘awry’ in their bodily functioning. Sensations are routinely-experienced bodily phenomena [37]. Individuals regularly cognitively grapple with unusual bodily sensations to interpret whether or not they are ‘normal’, or whether they transcend bodily equilibrium to become the subject of attention [38,39]. An individual’s assessment of sensation acceptability and their categorisation of certain sensations as ‘symptoms’ is informed by a number of factors; their perceived vulnerabilities, how long the abnormal sensation remains, the impact of the sensation on daily functioning and the creation of symptoms through social interaction, with the individual seeking guidance and advice from others about bodily experiences [38]. Embodied experiences are shaped by ‘local biologies’ [40], as the different physical and social aspects of an individual’s life influence how they experience, understand and respond to different bodily sensations, grounded within their specific socio-cultural and environmental context.

To frame examinations of people’s help-seeking in the context of ‘cancer symptoms’ is therefore problematic, as not only do they experience and respond to sensations and symptoms free of biomedical labelling, but do so in specific socio-cultural contexts informed by history, place, society and bodily expectations. In order to understand the factors that may influence the timing of help-seeking for cancer symptoms among people in rural areas, we need to take a step back and begin to examine how embodied sensations are experienced, created, defined and understood, both pre and post a cancer diagnosis.

## 3. Rural Locations

Locating symptomatic experiences within their rural environments is central to developing a meaningful understanding of how people experience and respond to symptoms. Existing research largely fails to reflect, or reflect upon, the context and complexity of rural environments. The mainly epidemiological studies to date have categorised place of residence (e.g., ‘rural’ vs ‘urban’). The distinction between rural and urban is usually based on travel time to health care services, settlement size, or population density (or a combination of these), with only a few studies even considering different degrees of rurality in their analyses [3]. This approach also constructs the ‘rural’ as the binary opposite of the ‘urban’. However, the ‘rural’ does not exist as a singular category. We know that the characteristics of ‘rural’ differ vastly between, and within, places and populations. This reductionist categorical treatment of patients’ place of residence in ‘big data’, seriously hinders our ability to understand and address the causes of poorer cancer outcomes in rural populations [41], preventing us from unpicking the complex interplay between embodied experience, rural environments and cancer pathways and outcomes.

Rural populations are differentially affected by issues of poverty, gentrification, ageing populations, in/out migration, occupational hazards, notions of rural masculinity and femininity, (health) service provision, and geographical and social isolation [2,42,43], all of which influence people’s lived experiences. Importantly, rural populations are dynamic, with the diversity of rural experience, and therefore the diversity of rural health and illness experiences, deeply influenced by globalisation [2]. Examinations of the body in rural spaces have largely focused on the interaction between healthy bodies and nature, as well as the gendered rural body. Rural bodies are often conceived of as ‘fit’ and ‘competent’, and rural spaces constitute environments conducive to ‘wellness’ and in which bodies can be more sensorially fulfilled than in urban environments [43]. Examinations of gendered rural bodies have shown that rural masculinity, particularly centred around the ‘strong farmer’ stereotype, is associated with health stoicism, while the focus on women’s bodies in rural environments has centred on the links between female bodies and nature, cycles and (re)production [18,43].

When it comes to accessing health care services, we know that distance to the nearest GP [18], poor access to transport [44], a tendency towards infrequent consultation [21], and differences in GP-patient relationships [23] have all been identified as influential in the decision-making of people living in rural areas. What remains unknown, however, is how local socio-cultural factors, such as poverty, lay beliefs about health and illness, the constraints of various rural occupations and caring responsibilities, and the effects of in/out migration, influence symptom appraisal and decision-making processes for both rural patients and rural health care practitioners. Whilst rural environments can pose different problems to urban environments in the experience and enactment of illness, the heterogeneity of rural environments means that these experiences are complex and contrasting.

## 4. A New Research Agenda

The time has come to reimagine how rural cancer inequality is conceived, examined and addressed, by acknowledging that rural bodies are not passive vessels, but are relational and territorialised [43]. In order to identify the underlying causes of rural cancer inequalities and address the demonstrably poorer survival rates experienced by rural populations, we need to develop an understanding of the contextual factors that shape how symptoms are experienced and acted upon, and how cancer-related decisions are made, both within and across a range of diverse rural environments.

Whilst analyses of large-scale datasets allow us to describe what is happening among rural populations, qualitative research provides us with different types of knowledge, that can help us to understand how and why inequalities exist and the contexts in which they arise [45]. For, ‘we can know about a world by describing it from the outside. Yet to understand what living in this world means, we need to learn from the inside’ [46]. The ability to understand people’s lived experience is one of the greatest assets of qualitative research, which grounds information on events and experiences within an appreciation of the wider personal, social, cultural and geographical contexts in which they occur.

Research into rural cancer inequalities needs to be grounded in detailed and nuanced understandings of the specific characteristics of, and interaction between, different rural sociocultural contexts, alongside the experience of embodied sensations, symptoms, illness, interactions with health care services and systems, and associated decision-making. Undertaking community-based research in a range of different rural environments and communities will be central to developing the foundational understandings of embodied rural cancer experiences needed to begin to deconstruct existing categories and reframe research questions. Knowledge derived from such approaches can illuminate our existing understanding of rural cancer inequalities, combining to produce a coherent body of knowledge capable of both describing and explaining this phenomenon. Such a synthesis can also identify what can be done to address rural cancer inequalities, and, importantly, where efforts should be targeted.

Qualitative research can, at times, be overlooked in relation to its capacity to enact meaningful change; however, given the multidimensional nature of ‘health problems’, the integration of multidisciplinary approaches is of great value [47]. The rich, in-depth understandings often generated through qualitative research of specific ‘health problems’, and the contexts in which they occur, can provide powerful evidence to inform and instigate policy-level interventions and legislative change [48]. In order to bring about change that is meaningful for rural populations, we must reflect upon, and make explicit in our analysis and reporting of qualitative research, the characteristics of the rural environments in which we locate our inquiry into diagnostic, treatment and follow-up pathways and cancer experiences of people living in rural settings. Involving people from a diverse range of communities in research endeavours is also vital, in order to design research that is feasible and appealing to rural populations, generate findings that have applicability across a diverse range of rural communities, and develop interventions that have the potential to improve survival for rural cancer patients.
